# The Effect of a *Bacillus*-Based Probiotic on Sow and Piglet Performance in Two Production Cycles

**DOI:** 10.3390/ani13203163

**Published:** 2023-10-10

**Authors:** Magdalena Mazur-Kuśnirek, Krzysztof Lipiński, Jens Noesgaard Jørgensen, Lea Hübertz Birch Hansen, Zofia Antoszkiewicz, Romuald Zabielski, Paweł Konieczka

**Affiliations:** 1 Department of Animal Nutrition, Feed Science and Cattle Breeding, University of Warmia and Mazury in Olsztyn, 10-718 Olsztyn, Poland; krzysztof.lipinski@uwm.edu.pl (K.L.); zofia.antoszkiewicz@uwm.edu.pl (Z.A.); 2 Chr. Hansen A/S, Animal and Plant Health & Nutrition, 2970 Hoersholm, Denmark; dkjjo@chr-hansen.com (J.N.J.); dkleha@chr-hansen.com (L.H.B.H.); 3 Center of Translational Medicine, Warsaw University of Life Sciences, ul. Nowoursynowska 100, 02-797 Warszawa, Poland; romuald_zabielski@sggw.edu.pl; 4 Department of Poultry Science, University of Warmia and Mazury in Olsztyn, 10-719 Olsztyn, Poland; p.konieczka@ifzz.pl

**Keywords:** probiotic, sow, piglets, performance, fecal score, fecal microbiota

## Abstract

**Simple Summary:**

Living microorganisms, known as probiotics, provide health benefits to the host and improve livestock performance. *Bacillus* strains are most commonly used as probiotics. In the present study, sows and piglets were fed diets supplemented with a probiotic containing *Bacillus subtilis* and *B. amyloliquefaciens* during two production cycles. Dietary probiotic supplementation improved the performance of sows and contributed to higher birth weight and weaning weight of piglets in two cycles.

**Abstract:**

The aim of this study was to assess the impact of Bacillus-based probiotic diets on reproduction performance, fecal scores, microflora, and economic factors in lactating sows and suckling piglets across two productive cycles. A total of 96 sows, reared in a continuous farrowing system for two full cycles, were divided into two groups: a control group and an experimental group. Sows were fed a basal diet without the probiotic or a diet supplemented with viable bacterial spores. At seven days of age, control group piglets were offered standard creep feed, whereas piglets in the experimental (probiotic) group received a diet containing the probiotic fed to their dams. Sows receiving probiotic-supplemented diets were characterized by significantly higher (*p* ≤ 0.05) average daily feed intake in lactation, lower (*p* ≤ 0.01) body weight (BW) loss during lactation, and reduced loss of backfat thickness as well as higher body condition score after lactation. Dietary probiotic supplementation increased (*p* ≤ 0.01) birth weight, total creep feed consumption, litter weight gain, and piglet weaning weight. The probiotic also improved (*p* ≤ 0.01) overall fecal scores, decreased total *E. coli* count on day seven and *Clostridium perfringens* count (trend) in sucking piglets. The total feed cost per weaned piglet was lower in the experimental (probiotic) group. Supplementing the diet with a probiotic containing *Bacillus* strains improved the reproductive performance of sows and the performance and health of piglets.

## 1. Introduction

In pig production, feed intake and body weight gain (BWG) can be compromised in three critical stages. The process of weaning can be highly stressful for piglets as it involves separation from sows and a transition from a milk-based to a plant-based diet [1]. These stressors can exert adverse effects on immunity and the gut microbiota balance in pigs [2], thus contributing to intestinal disorders, infections, and diarrhea [3].

In the past, antibiotic growth promoters (AGPs) as well as copper and zinc supplements were routinely added to the diets of weaned and growing piglets. However, increasing antimicrobial resistance posed a health threat to humans and animals [4], and on 1 January 2006, an EU-wide prohibition on the use of antibiotics as growth enhancers was implemented [5]. The above ban and the legislative restrictions on trace minerals that may be used in pig production [6] decreased animal performance and productivity by increasing feed intake per kg of BWG. The prevalence of intestinal disorders and, consequently, mortality rates increased in pig farms. The use of therapeutic antibiotics also increased in livestock production [7,8]. These problems have prompted the search for alternative growth promoters to minimize the adverse effects of piglet weaning. Numerous studies have demonstrated that pig diets can be supplemented with probiotics to balance gut microbiota, alleviate symptoms of intestinal diseases, and improve performance [9]. Research points towards carrying out interventions pre-weaning in order to have a beneficial impact on the health and performance of post-weaned pigs [10].

Probiotics are live microorganisms that modify and stabilize the gut microbiota and deliver health benefits to the host [11]. Probiotics can decrease intestinal pH, inhibit intestinal colonization by pathogens, stimulate immunity, and improve productivity [9,12]. Probiotic preparations contain one or more bacterial strains, as well as yeasts [3]. Research has shown that *Bacillus* spp. decrease diarrhea incidence, increase the absorptive surface area of intestinal villi, and improve nutrient digestibility, feed conversion ratio (FCR), and BWG [13,14]. According to many studies, *Bacillus subtilis* and *Bacillus amyloliquefaciens* have a positive impact on the health status and performance of sows [15,16] and the growth performance of piglets [17,18,19,20,21] over short- or medium-term periods. However, probiotics may exert varied and inconsistent effects. The efficacy of probiotic preparations is determined by the applied bacterial strains, inclusion rate, feed composition, and the animal’s age. Therefore, various combinations and concentrations of bacterial strains in the sow and piglet diets should be investigated. Furthermore, there are not many published studies investigating the efficacy of multi-strain probiotic preparations administered in feed to a very intensive production herd of DanBred sows and piglets over the course of two production cycles. It is of great importance that the administration of a probiotic product to sows carries a continuous beneficial impact on both reproductive performance and piglet vitality.

The research hypothesis postulates that a probiotic preparation consisting of *B. subtilis* (DSM25841) and *B. amyloliquefaciens* (DSM25840) added to diets for gestating and lactating sows during two cycles can improve their reproductive performance and the growth performance of piglets at weaning during both cycles. The objective of this study was to evaluate the effect of the *Bacillus*-based probiotic on performance parameters, fecal scores, and microbiota, as well as the results of economic analysis in sows and suckling piglets after long-term treatment (two cycles).

## 2. Materials and Methods

### 2.1. Experimental Design

The study was conducted at a pig farm located in northeastern Poland in Ławki. A total of 96 DanBred sows (primiparous and multiparous) were randomly divided into two groups (control and experimental). The sows had an average parity of 3.9 ± 1.98 in the first cycle and 4.0 ± 2.17 in the second cycle. They were reared in a continuous farrowing system for two full cycles. Gestating sows were housed in group pens and were fed a restricted feed ration (2.5–3.5 kg of feed daily). During lactation, sows were kept in individual stalls and fed increasing amounts of feed daily. The feed was evenly divided into three portions with approximately 6 h breaks throughout the day. The feeding curve assumed that on the first day of lactation, the sows consumed approximately 2 kg of feed, with an increase of 0.5 kg per day until the 10th day when they had ad libitum access to feed. Based on the leftovers that were left as well as the quantity, the daily ration of a sow was adjusted. The sows in the different treatment groups were housed in the same farrowing rooms. Throughout the study period, all pigs maintained their clinical health.

The sows were provided with mash-form diets. The experiment began when the sows in the gestation unit started receiving feed with probiotics, whereas the sows in the control group received feed without supplementation. The gestation diet was offered during the first 90 days of pregnancy, and the lactation diet was administered from day 90 of pregnancy until the end of lactation (Table 1). Gestation feed was also offered from weaning to service day in the second cycle. The control group (1—Control) animals received a basal diet without probiotics. The diets for the experimental group (2—Probiotic) were enriched with a probiotic preparation with calcium carbonate as a carrier, consisting of *B. subtilis* (DSM25841) and *B. amyloliquefaciens* (DSM25840) (1.1 × 10^9^ CFU/kg feed) supplied via top dressing in the amount of 400 g/ton of feed. Both strains were isolated from pig feces (Chr. Hansen A/S), and both have received a Qualified Presumed Safety certification, subject to confirmation of their non-toxic properties [22]. Pre-starter creep feed was offered to sucking piglets from day 7 after birth until weaning at 28 days of age (Table 2). In the control group, piglets were fed basal creep feed without probiotics, whereas piglets in the experimental group received creep feed containing probiotics fed to their dams.

Live body weight (BW), backfat thickness, and body condition scores (BCS) of sows were determined at the beginning of gestation, at farrowing, and at the end of lactation. Backfat thickness was assessed at the 10th rib, precisely 7.5 cm from one side of the backbone, using a digital backfat indicator (Lean-Meater, Renco Corp., Minneapolis, MN, USA). Sows’ body condition was visually assessed using a numeric rating scale ranging from 1 to 4 (1—very thin; 4—very fat) and was corroborated with backfat measurements. Daily feed intake was recorded for each sow during gestation and lactation. The wean-to-estrus interval (days) and non-productive days in sows were calculated.

The assessment included 88 litters in each production cycle. The following reproductive traits were recorded: number of piglets born alive, stillborns, mummies, and piglet losses. Piglet birth weight and litter birth weight were determined, and piglets were weighed at weaning on day 28 after birth. Litter weight gain and total creep feed intake were recorded.

Feces consistency of sows was evaluated daily from weeks 1 to 4, in ten sows from each group, on a four-point scale: 0—firm, dry pellets in a small hard mass, 1—soft, retaining its shape, 2—semi-liquid, and 3—liquid. The fecal scores of litters (3 piglets from 12 sows per group) were determined daily from birth until weaning, with the following scoring system: 0—no diarrhea, 1—diarrhea, 2—severe diarrhea.

### 2.2. Chemical Analysis

Feed samples underwent analysis to determine their composition, including dry matter (DM), crude ash, CP, ether extract (EE), and crude fiber (CF), according to [23].

For microbiological analysis, fresh fecal samples from 10 sows per group were collected at the entry to the farrowing unit, 7 days into lactation, and at the end of lactation. Fecal samples were also taken from 10 suckling piglets per group, at 7 days of age and on the day of weaning. All samples (10 g of fresh material each) were collected into sterile glass bottles (250 mL), and each sample was diluted in 90 mL of sterile saline water (0.85% NaCl).

Enumeration of *Lactobacillus* spp., spore-forming (*Bacillus* spp.), and *Clostridium* spp. Bacteria.

The surface plate technique was used to detect four groups of microorganisms. Portions (100 μL) of the respective sample dilutions (from 10–1 to 10–12) were transferred to plates and incubated at 37 °C for 48 h. For the enumeration of *Bacillus* spp., samples were preincubated at 80 °C for 15 min. To enumerate *Clostridium* spp., samples were meticulously prepared within anaerobic conditions and then cultured inside an anaerobic jar utilizing the Anaerocult system (MERCK).

The following culture media were used: TSA (Triptic Soy Agar- Merck, Darmstadt, Germany) for total bacteria and *Bacillus* spp., MRS (De Man, Rogosa, Sharpe agar, Merck, Darmstadt, Germany) agar for lactic acid bacteria, and TSC (Tryptose Sulfite Cycloserine- Merck, Darmstadt, Germany) for *Clostridium perfringens*.

Enumeration of *Escherichia coli*/*coliform* bacteria

The surface plate technique was employed to simultaneously detect total coliforms (TCC) and *E. coli* using Chromocult coliform agar (CCA- Merck, Darmstadt, Germany). Aliquots (100 μL) of the various sample dilutions (ranging from 10–1 to 10–12) were transferred onto plates and then incubated at 37 °C for 48 h. Pink colonies arising from the cleavage of salmon-galactoside by β-D-galactosidase were categorized as TCC, while dark blue colonies resulting from salmon-galactoside and X-glucuronide cleavage by β-D-galactosidase and β-D-glucuronidase were considered presumptive *E. coli* colonies.

Real-Time PCR for enterotoxigenic *Escherichia coli* (ETEC)

The presence of enterotoxigenic *E. coli* (ETEC) was detected by Real-Time PCR using the RotorGeneQ platform (Qiagen) based on the approach proposed by [24,25]. Two ETEC genes were identified in samples using the primers and probes described below:

I. Heat-labile enterotoxin (LT)
Primer1: LT-F TTCCCACCGGATCACCAA
Primer 2: LT-R CAACCTTGTGGTGCATGATGA
Probe: LT-P FAM-CTTGGAGAGAAGAACCCT-TAMRA
II. Heat-stable enterotoxin (ST)—porcine variant
Primer 1: STp-F TGAATCACTTGACTCTTCAAAA
Primer 2: STp-R GGCAGGATTACAACAAAGTT
Probe: STp-P FAM-TGAACAACACATTTTACTGCT-TAMRA
Real-Time PCR conditions: 10 min at 95 °C, 45 cycles of 15 s at 95 °C and 30 s at 58 °C.

### 2.3. Economic Analysis

The economic analysis was based on feed intake by gestating sows, lactating sows, and sucking piglets, as well as feed prices from February 2022. The total feed cost was determined for gestating sows, lactating sows, and the entire litter. The results were expressed per piglet and per kg of weaned piglet.

### 2.4. Statistical Analysis

Two diets were used as experimental factors for sows and litters: one with *Bacillus*-based probiotic and the other without it. The normality of the results was assessed using the Shapiro–Wilk test. Statistical analysis of the results was conducted using one-way analysis of variance (ANOVA) followed by Tukey’s test. Differences were regarded as significant at *p* ≤ 0.05, while 0.05 < *p* ≤ 0.10 was considered a near-significant trend. The results are presented in the Tables as the mean value with the pooled standard error (SEM). Calculations were performed in the STATISTICA 13.0 program (StatSoft, Krakow, Poland).

## 3. Results

Sow performance during two production cycles is presented in Table 3. Sows fed probiotic-supplemented diets were characterized by higher BW (trend) and backfat thickness (*p* ≤ 0.05) at the end of lactation, which contributed to lower losses in these parameters during lactation (*p* ≤ 0.01). Sows fed diets with the addition of *Bacillus* strains consumed more feed (*p* ≤ 0.05) during lactation, which most likely resulted in a higher BCS at the end of lactation (*p* ≤ 0.01). The analyzed probiotic had no effect on the remaining performance parameters of sows.

Litter performance during two production cycles is presented in Table 4. The addition of the tested probiotic to diets contributed to higher piglet weight at birth and weaning, and higher BWG and creep feed intake (*p* ≤ 0.01).

The fecal scores of sows and their litters in two production cycles are presented in Table 5. In the first week of the study, sows fed probiotic-supplemented diets had a higher (*p* ≤ 0.05) fecal score than control group sows. In piglets, fecal scores in weeks 1, 2 (*p* ≤ 0.01), and 3 (*p* ≤ 0.05), as well as overall fecal scores, were (*p* ≤ 0.01) better in the experimental group fed the probiotic.

The results of fecal microbiota analysis are presented in Table 6. The total spore count was higher (*p* ≤ 0.01) in fecal samples collected from sows fed probiotic-supplemented diets on day 90 of gestation, on day 7 of lactation, and at the end of lactation. On day 90 of gestation, the total lactic acid bacteria count tended to be higher in sows receiving the tested probiotic. At 7 days of age, the total spore count was higher (*p* ≤ 0.01), and the total *E. coli* count was lower (*p* ≤ 0.05) in piglets from the experimental group (probiotic). At weaning, piglets fed probiotic-supplemented diets were characterized by higher total spore count (*p* ≤ 0.05), a tendency of higher total lactic acid bacteria count, and a tendency of lower total *Clostridium perfringens* count.

The results of the economic analysis are presented in Table 7. An analysis of feed intake by sows and piglets and current feed prices revealed that the total feed cost per weaned piglet was lower in the experimental (probiotic) group.

## 4. Discussion

The purpose of this study was to assess the effectiveness of a *Bacillus*-based probiotic on performance parameters, fecal scores, and microbiota in both sows and their suckling piglets across two reproductive cycles. Sows are exposed to numerous stressors such as gestation, parturition, lactation, and piglet weaning, which may induce gut microbiota dysbiosis and negatively affect the health status and reproductive performance of the sows [26]. Therefore, sow and piglet diets can be supplemented with probiotics that can restore the intestinal microbial balance and alleviate the stress associated with gestation and lactation [27]. In the present study, BW and backfat losses were lower in sows administered probiotic-supplemented diets during lactation than in control group sows. In consequence, sows receiving a *Bacillus*-based probiotic were characterized by having a higher feed intake and BCS at the end of lactation. Other authors also found that increased intake of feed with the addition of probiotics improved the overall body condition of sows and reduced their BW loss, thus decreasing energy mobilization during lactation and backfat loss [28,29]. The body condition score of the sow at the end of pregnancy depends mainly on their BCS just after the preceding weaning, as well as their nutrient utilization during gestation [29]. Probiotics can increase the absorptive surface area of the intestinal mucosa, improve apparent nutrient digestibility [30,31], and increase the serum concentrations of cholesterol and total lipids [13], which indirectly contributes to reducing backfat loss and improving BCS in sows.

Nutrient digestibility and absorption in gestating and lactating sows affect the number of piglets born alive, as well as litter weight at birth and weaning [32]. Dietary probiotic supplementation improves nutrient utilization, which may increase milk yield and litter size [26]. In the current study, a probiotic preparation added to sow diets and creep feed for piglets contributed to higher litter weight at birth and weaning, as well as higher feed intake and BWG in piglets. Creep feed intake by piglets in early life stages supports gastrointestinal tract development by contributing to the renewal of small intestinal epithelium, modifying the production of brush border enzymes, and enhancing gut function, including nutrient absorption [33]. Probiotics affect small intestinal structure, and they can increase absorptive surface area by increasing the height of intestinal villi, thus improving nutrient digestibility [34]. Similar results to the ones observed in this study were reported by Alexopolus et al. [13] and Kritas et al. [29], who noted higher feed intake and weaning weight in piglets whose mothers were fed diets supplemented with a probiotic containing viable spores of *B. licheniformis* and *B. subtilis* or *B. subtilis* alone. In contrast, Wang et al. [35] and Jørgensen et al. [36] demonstrated that *Bacillus*-based probiotics had no influence on feed efficiency.

Weaning is one of the most stressful periods in a piglet’s life, which is often associated with decreased feed intake, lower BWG, and the risk of pathogen growth and proliferation [33] due to incomplete development of intestines and the immune system in young piglets [37,38]. The gut microbiota of sows participates in gastrointestinal tract colonization and, indirectly, immune function stimulation in newborn piglets [30,39,40]. During the period from birth until weaning, piglets are vulnerable to the colonization of their guts by pathogenic bacteria such as *Salmonella*, *E. coli*, *Clostridium perfringens*, as well as parasites including *Isospora* or *Cryptosporidium*, and viruses like coronaviruses and rotaviruses. These pathogens can lead to diarrhea and a decrease in the BWG. *Bacillus*-based probiotics are recommended as feed additives for young piglets, and their effectiveness in decreasing the count of pathogenic bacteria and improving fecal microbiota composition has been well documented [13,41,42]. In the present study, the fecal scores during the first week after farrowing were lower in sows receiving a *Bacillus*-based probiotic. In turn, the scores of fecal samples collected from piglets improved in response to dietary probiotic supplementation during the first three weeks of the experiment. The probiotic also decreased total *E. coli* count in 7-day-old piglets and tended to decrease *Clostridium perfringens* count in piglets at weaning.

In this study, the tested probiotic preparation contributed to improving the profitability of piglet production by reducing the total feed costs per kg of weaned piglet, relative to the control group (2.05 vs. 2.13 euro/kg). However, the ultimate results depend on feed prices, litter size, and the BW of animals.

## 5. Conclusions

The addition of a *Bacillus*-based probiotic to diets for gestating and lactating sows and their piglets during two production cycles improved their reproductive performance and contributed to an increase in litter weight at birth and weaning, thus increasing economic profitability. Dietary probiotic supplementation improved the fecal scores and fecal microbiota composition of piglets in two production cycles.

## Figures and Tables

**Table 1:** Composition, nutrient content, and energy value of sow diets.

Specification		Gestating Sows	Lactating Sows
Ingredient			
Barley	%	20.5	23.5
Triticale	%	12.5	10.0
Wheat	%	-	10.0
Oat	%	10.0	5.0
Corn	%	10.0	13.0
Wheat bran	%	20.0	3.5
Soybean meal	%	6.5	20.0
Sugar beet pulp	%	10.0	2.0
Apple meal (by-product from juice production)	%	4.0	5.0
Soybean oil	%	0.5	2.0
* Premix—Gestating sow 6%	%	6.0	-
** Premix—Lactating sow 6%	%	-	6.0
Nutrient content and energy value			
Metabolizable energy (ME)	MJ/kg	12.50	13.50
Crude protein (CP)	%	14.0	17.0
Lysine (Lys)	%	0.67	1.03
Methionine+ cystine (Met+ Cys)	%	0.51	0.64
Threonine (Thr)	%	0.53	0.68
Tryptophan (Trp)	%	0.17	0.21
Calcium (Ca)	%	0.95	0.85
Digestible phosphorus (d-P)	%	0.48	0.42
Sodium (Na)	%	0.22	0.22

* Composition of the premix for gestating sows: 12.5% Ca, 3.6% P; 3.2% Na; 1.2% Lys; 0.3% Met; 1% Thr; 1,800,000 IU vit. A, 33,340 IU vit. D3, 2000 mg of vit. E; 27 mg of vit. K3, 27 mg of vit. B1, 72 mg of vit. B2, 54 mg of vit. B6, 0.45 mg of vit. B12, 54 mg of folic acid, 180 mg of pantothenic acid, 360 mg of niacin, 8 mg of biotin, 7000 mg of choline chloride, 1280 mg of Mn, 2400 mg of Zn, 2400 mg of Fe, 400 mg of Cu, 32 mg of I, 8 mg of Se, antioxidant; xylanase+ β-glucanase + phytase. ** Composition of the premix for lactating sows: 13.3% Ca, 4.5% P; 3.3% Na; 4% Lys; 1.1% Met; 1.6% Thr; 1 800,000 IU vit. A, 33,340 IU vit. D3, 2000 mg of vit. E; 27 mg of vit. K3, 27 mg of vit. B1, 72 mg of vit. B2, 54 mg of vit. B6, 0.45 mg of vit. B12, 54 mg of folic acid, 180 mg of pantothenic acid, 360 mg of niacin, 8 mg of biotin, 7000 mg of choline chloride, 1280 mg of Mn, 2400 mg of Zn, 2400 mg of Fe, 400 mg of Cu, 32 mg of I, 8 mg of Se, antioxidant; xylanase+ β-glucanase + phytase.

**Table 2:** Composition, nutrient content, and energy value of creep feed.

Specification		Creep Feed
Ingredient		
Barley	%	20.0
Wheat	%	20.0
Corn	%	10.0
* Concentrate	%	50.0
Nutritional value		
ME	MJ/kg	14.00
CP	%	19.0
Lys	%	1.62
Met + Cys	%	0.99
Thr	%	1.04
Trp	%	0.31
Ca	%	0.73
d-P	%	0.45
Na	%	0.29

* Composition of the concentrate: extruded full-fat soybeans; soybean concentrate; soybean flour; porcine blood plasma; porcine blood cells; potato protein; fish meal; whey; 12% lactose; 1.4% Ca, 0.85% P; 0.5% Na; 2.9% Lys; 1.25% Met + Cys; 1.75% Thr; 0.5% of Trp; 32,000 IU vit. A, 4000 IU vit. D3, 330 mg of vit. E; 6 mg of vit. K3, 12 mg of vit. B1, 16 mg of vit. B2, 16 mg of vit. B6, 0.1 mg of vit. B12, 8 mg of folic acid, 120 mg of pantothenic acid, 80 mg of niacin, 0.4 mg of biotin, 1400 mg of choline chloride, 80 mg of Mn, 280 mg of Zn, 160 mg of Fe, 280 mg of Cu, 2.4 mg of J, 0.6 mg of Se, antioxidant; xylanase+ β-glucanase + phytase.

**Table 3:** Sow performance in two production cycles.

Item	1-Control	2-Probiotic	SEM	*p*-Value
Number of sows	48	48		
Average parity	3.9	4.0		
Body weight (kg)				
- entry to gestation unit	254.72	257.78	3.592	0.671
- entry to farrowing unit	312.97	316.69	3.410	0.586
- end of lactation	277.23 ^x^	289.69 ^y^	3.350	0.061
- loss during lactation, kg	35.73 ^a^	26.99 ^b^	0.757	<0.001
Backfat thickness (mm)				
- entry to gestation unit	16.23	16.40	0.258	0.737
- entry to farrowing unit	20.07	20.48	0.259	0.437
- end of lactation	16.39 ^a^	17.49 ^b^	0.251	0.027
- loss during lactation, mm	3.68 ^a^	2.99 ^b^	0.096	<0.001
Body Condition Score				
- entry to gestation unit	2.68	2.75	0.049	0.502
- entry to farrowing unit	3.23	3.35	0.040	0.122
- end of lactation	2.59 ^a^	2.83 ^b^	0.042	0.003
- change during lactation	0.65	0.52	0.039	0.112
Average daily feed intake, kg				
- gestation	3.21	3.20	0.008	0.472
- lactation	6.54 ^a^	6.75 ^b^	0.043	0.015
Farrowing rate, %	91.25	92.50	2.167	0.774
Wean-to-estrus interval, days	5.00	4.89	0.088	0.523
Non-productive days	10.63	7.78	0.969	0.141

^a^/^b:^ *p* ≤ 0.05; ^x^/^y^ 0.05 < *p* ≤ 0.10.

**Table 4:** Litter performance in two production cycles.

Item	1-Control	2-Probiotic	SEM	*p*-Value
Number of litters	88	88		
Number of piglets	1406	1432		
Litter size, n/litter				
total piglets born	19.00	19.21	0.270	0.702
piglets born alive	17.15	17.46	0.244	0.516
stillborn piglets	1.51	1.43	0.123	0.729
mummies	0.34	0.32	0.054	0.822
intrauterine growth-restricted (IUGR) piglets	2.39	2.15	0.147	0.408
after cross-fostering	14	14	-	-
Alive at 7 days of age, n/litter	12.81	12.88	0.040	0.356
Weaned, n/litter	12.61	12.77	0.051	0.117
Mortality before weaning, %	9.93	8.80	0.363	0.117
Piglet age at weaning, days	25.49	25.61	0.084	0.471
Piglet BW, kg				
birth weight of live piglets	1.26 ^a^	1.33 ^b^	0.013	0.002
individual piglet weight after cross-fostering	1.51	1.48	0.016	0.443
individual piglet weight at weaning	6.47 ^a^	6.80 ^b^	0.061	0.006
Litter weight, kg				
at birth	21.38 ^a^	22.90 ^b^	0.275	0.005
at cross-fostering	21.11	20.76	0.016	0.443
at weaning	81.59 ^a^	86.76 ^b^	0.855	0.002
Litter weight uniformity at birth	78.34	78.60	0.293	0.658
Litter weight uniformity at weaning	85.96	86.36	0.244	0.409
Litter weight gain				
total litter weight gain (from cross-fostering to weaning), kg	60.48 ^a^	66.00 ^b^	0.778	<0.001
average daily gain of piglets (from cross-fostering to weaning), g	194.87 ^a^	207.63 ^b^	2.174	0.003
Total creep feed consumption, kg	5.12 ^a^	5.53 ^b^	0.045	<0.001

^a^/^b^: *p* ≤ 0.05.

**Table 5:** Fecal scores in two production cycles.

Item		1-Control	2-Probiotic	SEM	*p*-Value
Fecal scores—sows, mean					
week	1	0.09 ^a^	0.20 ^b^	0.027	0.042
	2	0.20	0.24	0.032	0.452
	3	0.37	0.32	0.038	0.523
	4	0.44	0.42	0.042	0.770
overall		0.27	0.29	0.020	0.597
Fecal scores—litter, mean					
week	1	0.71 ^a^	0.32 ^b^	0.054	<0.001
	2	0.57 ^a^	0.27 ^b^	0.046	<0.001
	3	0.54 ^a^	0.33 ^b^	0.049	0.033
	4	0.40	0.40	0.041	1.000
overall		0.55 ^a^	0.33 ^b^	0.030	<0.001

^a^/^b^: *p* ≤ 0.05. The fecal scoring system of sows: 0—firm, dry pellets in a small hard mass, 1—soft, retaining its shape, 2—semi-liquid, and 3—liquid. The fecal scoring system of litters: 0—no diarrhea, 1—diarrhea, 2—severe diarrhea.

**Table 6:** Fecal bacterial enumeration in sows and piglets in two production cycles.

Item	1-Control	2-Probiotic	SEM	*p*-Value
Entry to farrowing unit (sows)				
Total spore count, CFU/g	9.37 × 10^4 a^	1.20 × 10^6 b^	1.57 × 10^5^	<0.001
Total lactic acid bacteria count, CFU/g	3.69 × 10^7 x^	2.09 × 10^8 y^	5.22 × 10^7^	0.099
Total *E. coli* count, CFU/g	2.51 × 10^8^	6.69 × 10^7^	7.02 × 10^7^	0.193
ETEC, detected/sample	4/20	4/20		
Total *Clostridium perfringens* count, CFU/g	3.67 × 10^6^	3.67 × 10^6^	1.21 × 10^6^	0.999
7 days into lactation (sows)				
Total spore count, CFU/g	1.98 × 10^5 a^	1.44 × 10^7 b^	2.91 × 10^6^	0.013
Total lactic acid bacteria count, CFU/g	5.48 × 10^7^	2.28 × 10^8^	1.07 × 10^8^	0.427
Total *E. coli* count, CFU/g	4.23 × 10^9^	3.46 × 10^9^	9.63 × 10^8^	0.697
ETEC, detected/sample	1/20	2/20		
Total *Clostridium perfringens* count, CFU/g	1.01 × 10^6^	1.62 × 10^5^	3.28 × 10^5^	0.201
End of lactation (sows)				
Total spore count, CFU/g	3.87 × 10^4 a^	2.02 × 10^6 b^	2.63 × 10^5^	<0.001
Total lactic acid bacteria count, CFU/g	7.20 × 10^7^	2.66 × 10^8^	8.87 × 10^7^	0.281
Total *E. coli* count, CFU/g	2.94 × 10^8^	2.91 × 10^8^	5.64 × 10^7^	0.979
ETEC, detected/sample	0/20	0/20		
Total *Clostridium perfringens* count, CFU/g	3.77 × 10^6^	4.10 × 10^5^	1.18 × 10^6^	0.890
7 days of age (litter)				
Total spore count, CFU/g	6.20 × 10^4 a^	1.83 × 10^5 b^	2.37 × 10^4^	0.009
Total lactic acid bacteria count, CFU/g	8.74 × 10^7^	2.03 × 10^8^	3.90 × 10^7^	0.140
Total *E. coli* count, CFU/g	2.64 × 10^8 a^	1.21 × 10^8 b^	3.54 × 10^7^	0.041
ETEC, detected/sample	1/20	2/20		
Total *Clostridium perfringens* count, CFU/g	1.26 × 10^8^	1.99 × 10^7^	3.50 × 10^7^	0.130
Weaning (litter)				
Total spore count, CFU/g	2.90 × 10^5 a^	1.69 × 10^6 b^	3.36 × 10^5^	0.035
Total lactic acid bacteria count, CFU/g	7.19 × 10^7 x^	5.11 × 10^8 y^	1.23 × 10^8^	0.073
Total *E. coli* count, CFU/g	1.36 × 10^8^	1.04 × 10^8^	3.68 × 10^7^	0.671
ETEC, detected/sample	2/20	1/20		
Total *Clostridium perfringens* count, CFU/g	1.49 × 10^7 x^	2.09 × 10^6 y^	3.64 × 10^6^	0.078

^a^/^b^: *p* ≤ 0.05; ^x^/^y^ 0.05 < *p* ≤ 0.10.

**Table 7:** Economic analysis in two production cycles.

Treatment	1-Control	2-Probiotic *
Total gestation feed intake, kg	374.1	372.97
Total lactation feed intake, kg	166.89	172.93
Total creep feed consumption, kg	5.12	5.53
Gestation feed cost, EUR/kg	0.3000	0.3036
Lactation feed cost, EUR/kg	0.3500	0.3536
Creep feed cost, EUR/kg	0.6700	0.6736
Total gestation feed cost, EUR/sow	112.25	113.23
Total lactation feed cost, EUR/sow	58.41	61.15
Total creep feed cost, EUR/litter	3.43	3.73
Total feed cost, EUR/litter	174.09	178.11
Piglets weaned per litter, n	12.61	12.77
Litter weight at weaning, kg	81.59	86.76
Total feed cost, EUR/piglet	13.81	13.95
Total feed cost, EUR/kg of weaned piglet	2.13	2.05

* Probiotic cost, dosage 400 g/t, probiotic cost—9.00 EUR/kg of product, probiotic cost—3.6 EUR/t of feed, 0.0036 EUR/kg of feed.

## Data Availability

The data presented in this study are available from the corresponding author.
